# Inhibition of Human Lung Cancer Cell Proliferation and Survival by Post-Exercise Serum Is Associated with the Inhibition of Akt, mTOR, p70 S6K, and Erk1/2

**DOI:** 10.3390/cancers9050046

**Published:** 2017-05-08

**Authors:** Nigel Kurgan, Evelyn Tsakiridis, Rozalia Kouvelioti, Jessy Moore, Panagiota Klentrou, Evangelia Tsiani

**Affiliations:** 1 Department of Health Sciences, Brock University, St. Catharines, ON L2S 3A1, Canada; nigel.kurgan@brocku.ca (N.K.); tsakire@mcmaster.ca (E.T.); rozalia.kouvelioti@brocku.ca (R.K.); jmoore6@brocku.ca (J.M.); nklentrou@brocku.ca (P.K.); 2 Centre for Bone and Muscle Health, Brock University, St. Catharines, ON L2S 3A1, Canada

**Keywords:** lung cancer, post-exercise serum, proliferation, survival, Akt, Erk1/2

## Abstract

Non-small cell lung cancer (NSCLC) accounts for 85% of all lung cancer cases, and for the most cancer-related deaths. The survival pathway of Akt, its downstream effectors, the mammalian target of rapamycin (mTOR) and ribosomal protein S6 kinase (p70 S6K), and the Ras-extracellular signal-regulated kinase (Erk1/2) pathways are activated in cancer leading to cell survival and growth. Thus, approaches that inhibit these signaling molecules may prove useful in the fight against lung cancer. Exercise is associated with health benefits and a limited number of studies indicate that serum from physically active individuals inhibit mammary and prostate cancer cell growth. In this study, we examined the effects of post exercise serum on proliferation, survival, and signaling cascades of human NSCLC cells. Blood was collected from male subjects prior to, 5 min, 1 h, and 24 h after a single bout of high intensity interval exercise on a cycle ergometer. Exposure of NSCLC cells to post exercise serum resulted in the inhibition of cell proliferation and survival, as well as significant reduction of phosphorylated/activated Akt, mTOR, p70 S6K, and Erk1/2 levels compared to cells treated with serum taken pre-exercise. Our data suggest that post exercise serum has anti-cancer properties in lung cancer and deserves further systematic investigation in animal models.

## 1. Introduction

Lung cancer accounts for the most cancer-related deaths globally and Non-Small Cell Lung Cancer (NSCLC) represents 85% of all lung cancer cases [1]. NSCLC is aggressive, develops resistance to conventional chemotherapy and/or radiation treatment, and fewer than 15% of patients reach a 5-year survival [1]. These statistics point to a need to develop effective strategies for NSCLC prevention and treatment that will hopefully decrease incidence and mortality rates. The three main histological forms of NSCLC include adenocarcinoma (40%), squamous cell carcinoma (25–30%), and large cell carcinoma (10–15%) [2]. Adenocarcinomas account for the majority of NSCLC cases, and there is evidence of increasing incidence rates worldwide when compared to other lung carcinomas [3].

Increased proliferation, reduced apoptosis, and increased survival are the main characteristics of cancer cells, which are achieved by modulation of key signaling molecules/pathways [4]. The phosphatidylinositol 3-kinase (PI3K)-Akt and the Ras-extracellular signal-regulated kinase (Erk1/2) signaling cascades are activated by growth factor binding to cell surface receptors, leading to enhanced cell proliferation and survival [5,6]. Activated Akt leads to downstream activation of the mammalian-target of rapamycin (mTOR), which in turn activates ribosomal S6 kinase (p70 S6K), resulting in increased protein synthesis and proliferation [7,8,9]. Akt also leads to phosphorylation and degradation of the pro-apoptotic protein Bad resulting in the inhibition of apoptosis and enhanced survival [10]. Akt is a proto-oncogene and its expression and activation is increased in many cancers, including NSCLC, and it is involved in the resistance of cancer cells to chemotherapy and radiation treatment [6,11]. In addition to Akt, the Ras signaling molecule is frequently mutated/activated in cancer, including NSCLC [12,13,14], leading to downstream activation of Erk1/2 and resulting in cell proliferation, and chemo and radiation resistance [15]. Therefore, finding strategies of targeting/inhibiting the Akt and the Ras-Erk1/2 signaling cascades may be an effective approach to the prevention and treatment of lung cancer.

Regular exercise is associated with overall health benefits and reduced risk of many cancers [16], including prostate [17] and breast cancer [18]. Breast cancer patients participating in light aerobic exercise had increased survival compared to sedentary patients [19] and had almost 90% lower cancer mortality risk when the physical activity level was increased (running >1.8 metabolic equivalent of task (MET)/day) [20]. Although the association between exercise and lung cancer is not extensively investigated, regular exercise (>4 h/week) at a moderate intensity (>4.5 MET) has been shown to reduce the risk of NSCLC adenocarcinomas in men and women when compared to individuals exercising for less than 4 h/ week at low intensity (<4.5 MET) [21,22]. Furthermore, there appears to be a 20–40% risk reduction of lung cancer with regular exercise when compared to sedentary controls [23]. Despite this epidemiological evidence, the exact factors and mechanisms involved in the anti-cancer effects of exercise are not known.

In the present study, we investigated the effect of serum collected pre-exercise or post high intensity interval exercise (HIE) on human NSCLC cell proliferation, survival, and the effects on Akt, mTOR, p70 S6K, and Erk1/2 phosphorylation/activation and expression.

## 2. Results

**Figure 1:**
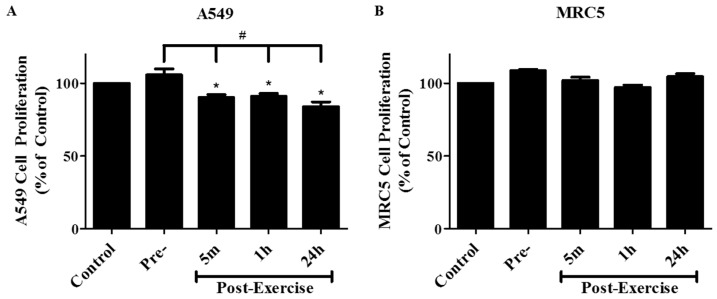
Effect of human post-exercise serum on A549 (**A**) and MRC5 (**B**) cell proliferation. Cells were exposed to media containing 10% FBS (control), 10% pre-exercise, 5 min, 1 h, or 24 h post-exercise serum for 72 h followed by fixing and staining with 0.5% crystal violet. The stain was solubilized and absorbance was read at 570 nm. Results are expressed as % of control and are the mean ± SEM of 4–6 independent experiments corresponding to serum from 4–6 different subjects. * *p* < 0.05 compared to control cells, ^#^ *p* < 0.05 compared to cells treated with pre-exercise serum, (ANOVA).

Human lung adenocarcinoma A549 cells were treated with either 10% FBS or 10% human serum collected pre-exercise, 5 min, 1 h, or 24 h post-exercise. Treatment of A549 cells with serum taken 5 min, 1 h, and 24 h post-exercise resulted in a significant inhibition of cell proliferation of 90.1 ± 4.2%, 91.0 ± 2.1%, and 84.1 ± 1.9%, respectively, when compared to the control FBS-treated cells, *p* < 0.05, (Figure 1A). Treatment of the cells with pre-exercise serum did not have any significant effect on cell proliferation. We also examined the effect of post-exercise serum on MRC5 normal lung fibroblasts. Exposure of MRC5 cells to post-exercise serum did not have any significant effect on cell proliferation (Figure 1B).

**Figure 2:**
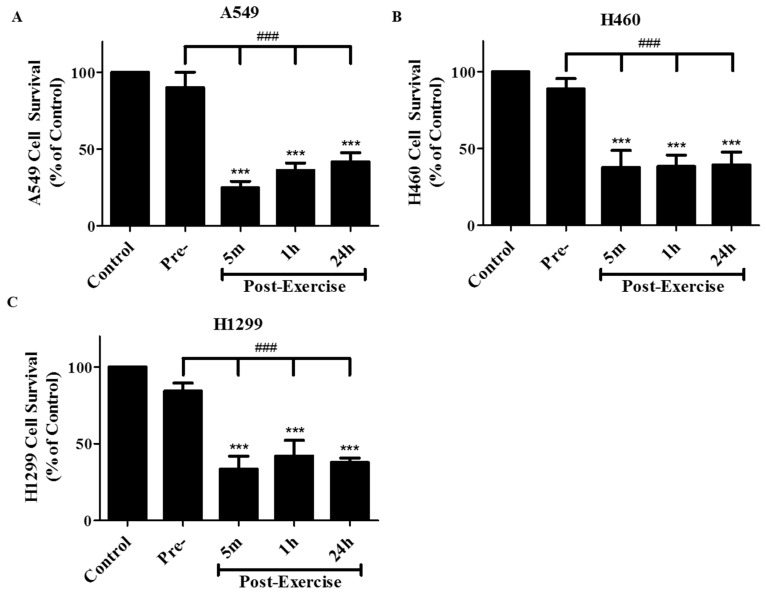
Effect of human post-exercise serum on clonogenic survival of A549 (**A**), H460 (**B**), and H1299 (**C**) cells. Cells were exposed for 7 days to media containing 10% FBS (control) or 10% human serum collected pre-exercise, 5 min, 1 h, or 24 h post exercise followed by fixing and staining with 0.05% methylene blue. Colonies of more than 50 cells were counted. Results are expressed as % of control and are the mean ± SEM of 6 independent experiments corresponding to serum from 6 different subjects. *** *p* < 0.001, compared to control cells, ^###^ *p* < 0.001 compared to cells treated with pre-exercise serum, (ANOVA).

The significant inhibition of cancer cell proliferation seen with post exercise serum prompted us to examine its effects on the ability of cancer cells to survive and form colonies. Exposure of A549 adenocarcinoma cells to 10% pre-exercise human serum did not have any significant effect on cell survival when compared to control cells exposed to 10% FBS-containing media (Figure 2). However, exposure of the cells to media containing 10% serum collected 5 min, 1 h, or 24 h post-exercise resulted in survival rates of 21.5 ± 2.9%, 33.9 ± 3.5%, and 35.8 ± 6.7%, respectively, compared to the control FBS-treated cells, *p* < 0.001, (Figure 2A). Exposure of the cells to media containing 10% post-exercise serum resulted in a significant inhibition of cell survival when compared to cells exposed to pre-exercise serum, *p* < 0.001, (Figure 2A). In addition to A549 cells we used the H460 and the H1299 lung cancer cells. Exposure of H460 cells to media containing 10% serum collected 5 min, 1 h, or 24 h post-exercise resulted in survival rates of 37.7%, 38.4% and 39.3%, respectively, compared to the control FBS-treated cells, *p* < 0.001, (Figure 2B). Similarly, exposure of H1299 cells to media containing 10% serum collected 5 m, 1 h, or 24 h post-exercise resulted in survival rates of 33.5%, 41.9%, and 37.7%, respectively, compared to the control FBS-treated cells, *p* < 0.001, (Figure 2C).

**Figure 3:**
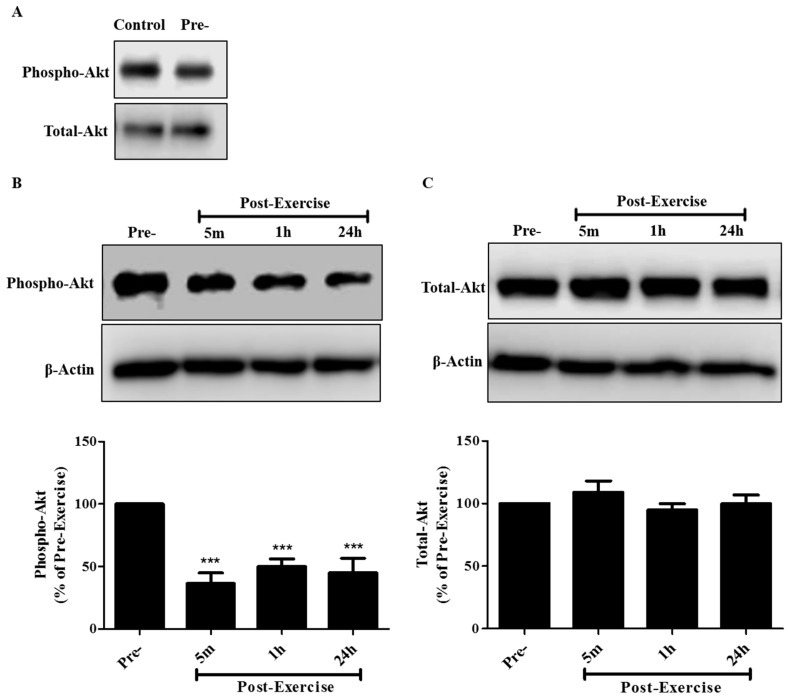
Effect of human post-exercise serum on Akt signaling in A549 NSCLC cells. Whole cell lysates were prepared from A549 cells exposed for 30 min to 10% FBS-containing media (control) or media containing 10% human serum taken pre-exercise, 5 min, 1 h, or 24 h post-exercise. Cell lysates (20 µg) were resolved by sodium dodecyl sulfate polyacrylamide gel electrophoresis (SDS-PAGE) and immunoblotted with specific antibodies against phospho or total Akt. (**A**–**C**) Upper panels: Representative immunoblots of phospho Akt, total Akt, and β-actin, used as a loading control. Lower panels: The densitometry of the bands was measured using ImageJ software and is expressed as a percentage of pre-exercise serum-treated cells. Results are the mean ± SEM, of 7 independent experiments corresponding to serum from 7 different subjects. *** *p* < 0.001, (ANOVA).

Next, we investigated the potential underlying mechanisms involved in the inhibition of proliferation and survival seen with post-exercise serum and examined the effect on the phosphorylation/activation of Akt, the protein that is often mutated/over activated in lung cancer [7,8,9]. The phosphorylation of Akt on serine 473 has been established as an indicator of Akt activity and therefore a phospho-specific antibody against this residue (Ser 473) was used. Exposure of the A549 cells to 10% human serum taken 5 min, 1 h, or 24 h post-exercise resulted in Akt phosphorylation levels of 36.6 ± 9.8%, 50.2 ± 7.6%, and 45.0 ± 13.8% respectively, when compared to the cells treated with serum taken pre-exercise (Figure 3B). Total Akt levels were not different across all treatments (Figure 3C). Total and phosphorylated Akt levels in cells treated with pre-exercise serum were the same as in cells exposed to media containing 10% FBS (Figure 3A), and based on this evidence the rest of the experiments were focused on comparing the effects of post-exercise serum to the effects of pre-exercise serum.

**Figure 4:**
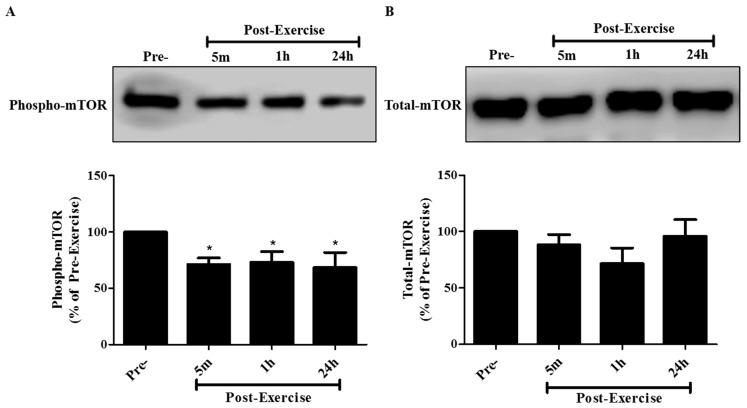
Effect of human exercise serum on mTOR signaling in A549 NSCLC cells. Whole cell lysates were prepared from A549 cells exposed for 30 min to media containing 10% serum taken pre-exercise, 5 min, 1 h, or 24 h post-exercise. Cell lysates (20 µg) were resolved by SDS-PAGE and immunoblotted with specific antibodies against (**A**) phospho or (**B**) total mTOR. Upper panels: Representative immunoblots of phospho mTOR, and total mTOR. Lower panels: The densitometry of the bands was measured using ImageJ software and is expressed as a percentage of pre-exercise serum-treated cells. Results are the mean ± SEM, of 7 independent experiments corresponding to serum from 7 different subjects. * *p* < 0.05, (ANOVA).

Activation of Akt leads to the activation of downstream signaling molecules, such as mTOR and p70 S6K, which lead to increased protein synthesis and increased proliferation and survival. We therefore examined the effect of post-exercise serum on these key regulators of proliferation and survival. Activation of mTOR was assessed by measuring phosphorylation levels on serine 2448, as it is an established indicator of mTOR activation. Incubation of A549 cells with 10% serum taken 5 min, 1 h, or 24 h post-exercise resulted in mTOR phosphorylation levels of 71.3 ± 5.8%, 73.1 ± 9.5%, and 68.4 ± 13.4%, respectively, when compared to the cells treated with serum taken pre-exercise (Figure 4A). Total mTOR levels were not different across all treatments (Figure 4B).

**Figure 5:**
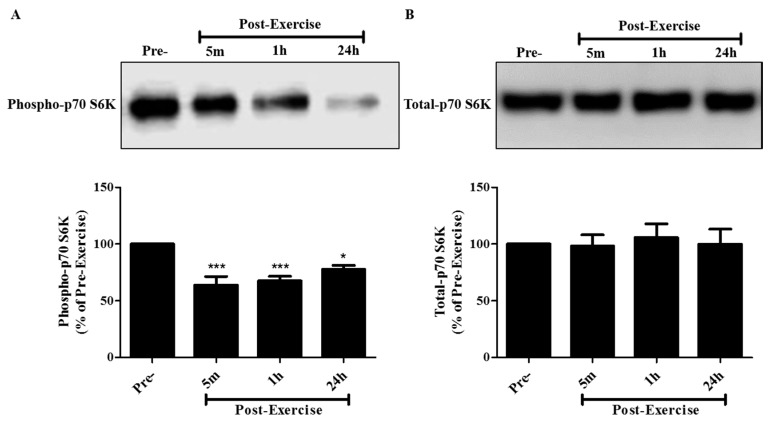
Effect of human exercise serum on p70 S6K signaling in A549 NSCLC cells. Whole cell lysates were prepared from A549 cells exposed for 30 min to media containing 10% serum taken pre-exercise, 5 min, 1 h, or 24 h post-exercise. Cell lysates (20 µg) were resolved by SDS-PAGE and immunoblotted with specific antibodies against (**A**) phospho or (**B**) total p70 S6K. Upper panels: Representative immunoblots of phospho p70 S6K and total p70 S6K. Lower panels: The densitometry of the bands was measured using ImageJ software and is expressed as a percentage of pre-exercise serum-treated cells. Results are the mean ± SEM, of 5 independent experiments corresponding to serum from 5 different subjects. * *p* < 0.05, *** *p* < 0.001, (ANOVA).

Similar to the effects seen on mTOR, exposure of the cells to media containing 10% post-exercise serum resulted in a significant inhibition of p70 S6K phosphorylation/activation on the threonine 389 residue. Incubation of A549 cells with 10% human serum taken 5 min, 1 h, or 24 h post-exercise resulted in p70 S6K phosphorylation levels of 63.8 ± 7.7%, 67.6 ± 4.0%, and 77.7 ± 3.5%, respectively, compared to cells treated with pre-exercise serum (Figure 5A). Total p70 S6K levels were not different across all treatments (Figure 5A).

**Figure 6:**
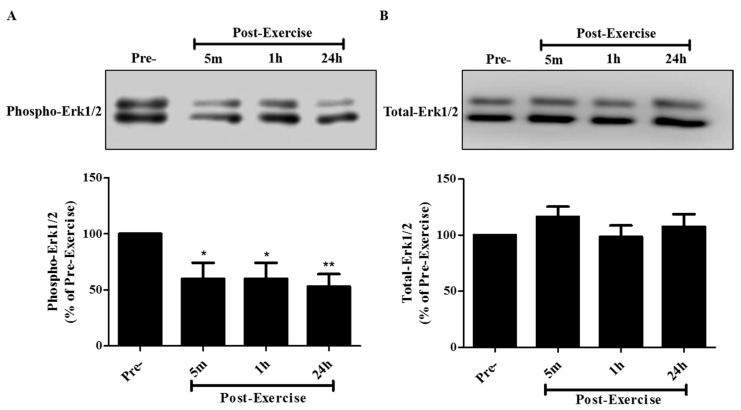
Effect of human exercise serum on Erk1/2 signaling in A549 NSCLC cells. Whole cell lysates were prepared from A549 cells exposed for 30 min to media containing 10% serum taken pre-exercise, 5 min, 1 h, or 24 h post-exercise. Cell lysates (20 µg) were resolved by SDS-PAGE and immunoblotted with specific antibodies against (**A**) phospho or (**B**) total Erk1/2. Upper panels: Representative immunoblots of phospho Erk1/2 and total Erk1/2. Lower panels: The densitometry of the bands was measured using ImageJ software and is expressed as a percentage of pre-exercise serum-treated cells. Results are the mean ± SEM, of 5 independent experiments corresponding to serum from 5 different subjects. * *p* < 0.05, ** *p* < 0.01.

The onco-protein Ras is activated in cancer leading to downstream activation in Erk1/2 that drives enhanced proliferation. Therefore, we wished to see if post-exercise serum influenced this signaling cascade and for this reason we examined Erk1/2 phosphorylation/activation on the threonine 202 and tyrosine 204 (Thr202/Tyr204) residues. Incubation of A549 cells with 10% human serum taken 5 min, 1 h, or 24 h post-exercise resulted in Erk1/2 phosphorylation levels of 60.0 ± 14.2%, 60.0 ± 14.2%, and 52.9 ± 11.2%, respectively, compared to the cells treated with pre-exercise serum (Figure 6A). Total Erk1/2 levels were not different across all treatments (Figure 6B).

**Figure 7:**
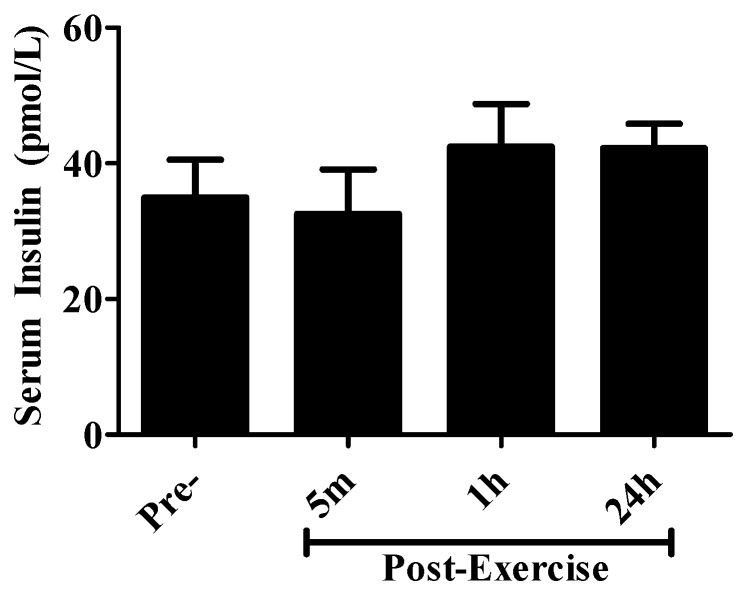
Insulin levels in human serum pre- and post-exercise. Insulin levels were quantified in serum taken pre-exercise, 5 min, 1 h, or 24 h post-exercise using a quantitative sandwich enzyme immunoassay. Absorbance was read at 450 nm. Results are expressed in pmol/L and are the mean ± SEM, of serum insulin levels from 10 different subjects.

Insulin is a strong growth factor; it directly activates the Akt-mTOR and the Ras-Erk signaling pathways and therefore we measured serum insulin levels to examine if the observed changes could potentially be explained by the changes in serum insulin. Serum insulin levels were not significantly changed (pre-exercise, 5 min, 1 h, or 24 h post-exercise: 34.9 ± 5.6, 32.5 ± 5.6, 42.4 ± 6.3, 42.2 ± 3.6 pmol/L, respectively) (Figure 7).

## 3. Discussion

**Figure 8:**
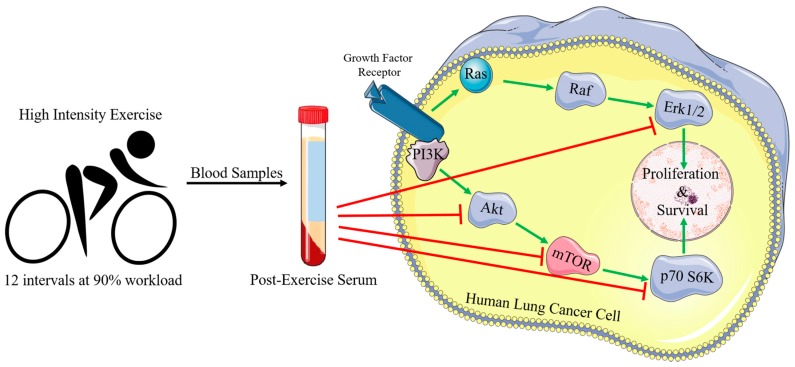
Potential mechanism of the inhibition of lung cancer cell proliferation and survival by post-exercise serum. Post-exercise serum inhibited Akt, mTOR, p70 S6K, and Erk1/2 phosphorylation/activation and resulted in significant inhibition of cell proliferation and survival.

In this study, we investigated whether serum collected from humans after an acute bout of high intensity exercise could lead to anti-cancer effects in vitro, and what signaling pathways might be affected. Our data are summarized in Figure 8 and illustrate that the treatment of lung cancer cells with serum taken 5 min, 1 h, and 24 h post-exercise leads to significant inhibition of cell survival and proliferation. Importantly, post-exercise serum did not have any significant effect on normal lung fibroblasts. This study is the first to show the inhibition of lung cancer cell proliferation and survival by post-exercise serum and is in agreement with previous studies showing similar effects in prostate [24] and breast [25] cancer cells. Comparable to our data, Rundqvist et al. (2013) showed that treating LNCaP prostate cancer cells with human serum taken immediately following 60 m of cycling lead to a 31% inhibition of cancer cell growth when compared to cells treated with serum taken pre-exercise [24]. A recent study found that serum taken 2 h following an acute bout of exercise from non-trained individuals reduced MCF-7 and MDA-MB-231 breast cancer cell viability significantly [25]. Furthermore, when MCF-7 mammary cancer cells were treated with serum taken immediately post-exercise (swimming) from mice, there was a significant inhibition of cell proliferation (52%) and increased caspase activity (54%), indicating apoptosis, compared to cells treated with serum taken from mice pre-exercise [26].

Apart from the effects of serum collected following an acute bout of exercise, there are studies indicating that serum from individuals participating in exercise programs regularly has anticancer effects in cancer cells in vitro, compared to serum from sedentary controls. The inhibition of prostate [27,28,29,30,31,32,33,34], mammary [35], and hepatocellular [36] cancer cell proliferation from serum taken following a chronic diet and exercise intervention was significant when compared to cells treated with serum taken from sedentary controls or pre-intervention.

Taken together, the above studies demonstrate that acute exercise as well as exercise training (chronic exercise) induce changes in serum factors that can lead to significant inhibition of cancer cell growth in vitro. Unfortunately, the exact serum factors contributing to the anticancer effects of exercise are not known. Exercise affects the levels of circulating hormones, growth factors, and cytokines, and in recent years contracting/exercising skeletal muscle has been recognized as an important tissue producing and releasing into the bloodstream myokines and other substances that affect the functionality of other tissues, resulting in health benefits [37]. The effect of exercise on serum factors and their subsequent contribution to exercises’ anti-cancer effects has been assessed in prostate [28,29,30,31,32] and breast [35] cancer cell lines. Individuals participating in regular exercise training had lower serum testosterone, estradiol, and insulin levels compared to sedentary controls, and when these hormones were added to the serum of men in the intervention/exercise group to match the higher levels in the serum from the sedentary group, the reduction in prostate cancer cell growth originally seen in the exercise group was attenuated [29]. We measured serum insulin and found no significant differences between the pre- and post-exercise levels, indicating that the observed effects are not due to insulin. Evidence indicates that regular chronic exercise resulted in decreased insulin-like growth factor-1 (IGF-1) and increased insulin-like growth factor binding protein-1 (IGFBP-1) serum levels [24,28,30,31,32,35]. When IGF-1 was added to post-intervention serum to match the IGF-1 levels in the pre-intervention serum, the inhibition in prostate cancer cell growth was attenuated [32]. This study concluded that diet and exercise intervention may help prevent clinical prostate cancer and be a treatment strategy during the early stages of development. Similarly, when IGFBP-1 was added to pre-intervention serum to match the levels seen in post-intervention serum, cell growth was reduced and apoptosis was induced [31]. Thus, IGF-1 and IGFBP-1 may play important roles in modulating the effect of exercise serum on cancer cell growth and survival.

Media from electrically-stimulated myotubes inhibited MCF-7 mammary cancer cell proliferation and induced apoptosis, an effect that was similar to the effects of post-exercise serum [26]. These beneficial effects were significantly reduced when neutralizing oncostatin M antibodies were used, suggesting that the exercising/contracting muscle is producing oncostatin M which then exerts significant anticancer effects [26]. However, the role of oncostatin M on cancer cell proliferation remains controversial as it has been proposed as both a tumor suppressor [38] and a tumor promotor [39]. Therefore, further research is required to better understand the effect of oncostatin M on cancer cell growth. Secreted protein acidic and rich in cysteine (SPARC) is shown to be significantly increased in human serum and mouse muscle immediately following acute exercise [40]. The reduction in tumorigenesis and increased caspase activity which occurred following acute exercise in azoxymethane-induced colon cancer in wild-type mice was not seen in SPARC-null mice, suggesting that SPARC may be a critical myokine involved in the anticancer effects of exercise [40]. Another myokine increased in serum after an acute exercise and shown to exert anticancer effects is irisin [41]. Irisin significantly decreased cell number, migration, and viability in MDA-MB-231 breast cells, without affecting normal breast cells, in part by enhancing caspase activity and suppressing NFkB activity. Irisin also augmented the cytotoxic effects of doxorubicin, an established chemotherapeutic agent, in breast cancer cells [41]. Additionally, there are several other myokines that have been proposed to have anti-cancer effects (reviewed in [37]).

Interestingly, serum collected 24 h post-exercise had a similar inhibitory effect on cancer cell survival, Akt, mTOR, p70S6K, and Erk phosphorylation, as serum collected 5 min and 1 h post-exercise. These data indicate that changes in serum factors, induced by exercise and responsible for its anticancer effects, persist 24 h post-exercise. We measured insulin levels and found no significant changes. Additionally, it should be noted that the serum used in the present study was assessed for cytokine levels previously [42]. IL-1α, IL-1β, IL-10, IL6, and TNF-α levels were found to be increased 5 min post-exercise and returned to baseline levels 1 h and 24 h post-exercise [42]. Even if we assume that myokines such as oncostatin M, SPARC, and irisin are contributing to the anticancer effects of post-exercise serum [37], we do not expect these molecules/proteins to be significantly elevated in 24 h post-exercise serum. Indeed, serum irisin levels were elevated immediately after a high intensity interval exercise and returned to baseline levels 1 h post-exercise [43]. Based on the limited evidence, we hypothesize that small, non-statistically significant, changes in different serum factors persist 24 h post-exercise which then contribute in a synergistic and/or additive manner to inhibit key signaling molecules and exert anticancer effects. Further investigation is required to determine the serum factors/myokines induced by exercise and responsible for the anticancer effects observed. We did not assess potential serum factors (besides insulin), because the focus of the present study was to elucidate the signaling mechanisms contributing to the anticancer effects of post-exercise serum. Despite the identification of several potential factors responsible for the anti-cancer effects of exercise, there are limited studies that have assessed the signaling pathways that are affected by post-exercise serum treatment of cancer cells. The inhibition of Akt, its downstream effectors, mTOR and p70 S6K, as well as the inhibition of Erk1/2 seen in our study is very important and represents a novel finding. Our study is the first to show significant inhibition of these pathways by post-exercise serum exposure in NSCLC cells. Our findings are supported by a previous study showing a reduction in Akt phosphorylation in mammary cancer cells treated with media from cultured adipocytes from high fat diet-fed animals performing voluntary exercise [44]. Activated Akt leads to the activation of mTOR and p70 S6K [7,8,9] and thus, the inhibition of Akt by post-exercise serum could be responsible for the inhibition of mTOR and p70 S6K seen in our study.

Our study showed that post-exercise serum was able to inhibit the activation of Erk1/2 in A549 cells, suggesting that this significant inhibition of Erk1/2 contributes to the inhibition in cell proliferation and survival that was seen. One previous study has also shown that serum taken from men following 3 weeks of low-fat diet and moderate aerobic exercise intervention was associated with a significant reduction of phosphorylated and total levels of Erk1/2 compared to pre-intervention serum in HepG-2 liver cells [36].

A549 cells harbour a mutation in the Ras GTPase protein (KRAS), which leads to constitutive activation of the Ras-PI3K-Akt signaling cascade and enhanced Ras-MAPK (Erk1/2) signaling. This cell line has high basal levels of Akt and Erk1/2 activation [45] and represents an aggressive form of NSCLC clinically. Importantly, both Akt and Erk1/2 activity were attenuated by post-exercise serum. Studies have found that the blocking activity of the PI3K/Akt axis using inhibitors such as AZD5363 (Akt) or LY294002 (PI3K) results in enhanced tumor sensitivity to chemotherapy agents and ionizing radiation [46]. There are currently clinical trials underway focused on the use of PI3K/Akt inhibitors to improve patient outcomes following developed resistance to anticancer therapies such as epidermal growth factor receptor (EGFR) tyrosine kinase inhibitors [5,47] (reviewed in [48]) which represent a first line of treatment for NSCLC patients. Perifosine is a novel oral inhibitor of Akt which is currently in phase 3 clinical trials and importantly, it has been shown to inhibit the phosphorylation of Akt and downstream mTOR and p70 S6K in various NSCLC cell lines [49]. Our study strongly supports further investigation of the anticancer effects of post-exercise serum, since it shows comparable effects to perifosine and other novel Akt inhibitors under investigation (reviewed in [6,50]). Post-exercise serum (1) may target Akt directly leading to the reduced mTOR/p70 S6K activity; (2) may target mTOR and p70 S6K directly or (3) may target molecules upstream of Akt such as PI3K or PDK1/2. 

Future studies should explore the effects of post-exercise serum on other signaling molecules, such as AMPK, p53, and caspases. Studies using animal models of cancer should be employed to investigate the potential risks/benefits of acute versus chronic exercise as well as which modes of exercise (i.e., cycling, running, or swimming) may be more beneficial than others. Furthermore, because exercise is already considered an important component of maintaining general health, clinical trials with cancer patients have recently begun, although their clinical benefits remain unclear [6,50], thus further research is needed.

## 4. Materials and Methods

### 4.1. Materials

Human A549, H460, and H1299 NSCLC cells and MRC5 normal lung fibroblasts were obtained from the American Type Culture Collection (ATCC). Cell culture (RPMI and DMEM) media, fetal bovine serum (FBS), trypsin, and antibiotic-antimycotic were from GIBCO (Burlington, ON, Canada). Total and phospho-specific antibodies Akt (S473), mTOR (S2448), p70 S6K (T389), Erk1/2 (T202, Y204), β-actin, and secondary anti-rabbit IgG HRP-linked antibodies were from Cell Signaling Technology via New England Biolabs (Mississauga, ON, Canada). Bovine serum albumin (BSA), leupeptin, PMSF, Na_3_VO_2_, glycerol, and dimethyl sulfoxide (DMSO) were purchased from Sigma (Oakville, ON, Canada). Clarity Western enhanced chemiluminescence substrate (ECL), sodium dodecyl sulfate (SDS), 30% acrylamide/bis solution 37 (5:1), ammonium persulfate (APS), Tween 20, tetramethylethylenediamine (TEMED), bromophenol blue, 2-mercaptoethanol, Biorad protein assay reagent, polyvinylidene difluoride (PVDF) membranes, molecular weight protein standards, and electrophoresis reagents were from BioRad (Hercules, CA, USA). Glycine was purchased from BioShop (Burlington, ON, Canada). Tris-HCl was purchased from Fisher chemical (Fair Lawn, NJ, USA). Skim milk was purchased from Great Value (Mississauga, ON, Canada). Triton X-100, EGTA, and sodium pyrophosphate were purchased form EMD (Gibbstown, NJ, USA). NaCl was purchased from ACP (Montreal, QC, Canada). EDTA was purchased from Caledon (Georgetown, ON, Canada). Bovine serum albumin was from Calbiochem (Gibbstown, NJ, USA).

### 4.2. Exercise Protocol and Blood Collection

Recreationally active male University students (21.8 ± 2.4 years of age; 76.63 ± 11.7 kg) were recruited to participate in a high intensity exercise (HIE) study previously described [42]. Participants were instructed to consume a good meal rich in carbohydrates and fluids approximately 2 h prior to arrival in the laboratory. In brief, the study consisted of three visits on separate days with two visits being on consecutive days. The first visit consisted of a 5 min warm-up session of cycling at 80 Watts (W), followed by the incremental test in which the resistance was increased by 15 W every minute. Participants cycled until exhaustion, and the maximum workload was recorded. On the second visit, participants performed a HIE trial based on the cycle ergometer at 90% of the maximum workload. The trial involved six 1-min high-intensity cycling intervals separated by six 1-min active rest periods. The HIE trial was initiated with a 4-min warm-up, and was followed by a cool-down period of 2–3 min that consisted of cycling at 70 W. All subjects were asked to refrain from exercise for at least 24 h prior to the HIE trial and between the third and last blood sample collection. Blood was drawn from the medial cubital vein at four time points: pre-exercise (pre-), 5 min post-exercise (5 min), 1 h post-exercise (1 h), and 24 h post-exercise (24 h) using 21 G BD Vacutainer needles and blood collection tubes. Samples sat at room temperature for 60 min to clot. Tubes were then centrifuged (Beckman Coulter Allegra 21R centrifuge) at 1000 *g* and 4 °C for 15 min. Serum was aliquoted into polypropylene microfuge tubes. The serum collected from the study participants was not pooled. Serum collected from the different subjects was stored at −80 °C until use.

### 4.3. Cell Culture and Treatment

A549, H460, and H1299 cells were grown in RPMI media and MRC5 cells were grown in DMEM media, both supplemented with 10% (*v*/*v*) FBS, and 1% (*v*/*v*) antibiotic-antimycotic solution in a humidified atmosphere of 5% CO_2_ at 37 °C. Cells were serum deprived for 3 h prior to immunoblotting to synchronize cells by arresting them in the G_0_/G_1_ phase before treatment with 10% of either FBS or serum taken pre-exercise, 5 min, 1 h, or 24 h post HIE. Serum from the different subjects was used to treat cells in triplicates.

### 4.4. Cell Proliferation Assay

The crystal violet cell proliferation assay was performed as described previously [51]. Briefly, A549 and MRC5 cells were seeded (1000 cells/well) in triplicate in 96-well plates, incubated with 10% FBS or human serum for 72 h, fixed, and stained with crystal violet dye. The following day the dye was solubilized and absorbance was read at 570 nm using a KC4 plate reader (Bio-Tek).

### 4.5. Clonogenic Survival Assay

Clonogenic survival assays were performed as described previously [51]. Briefly, A549, H460, and H1299 cells were seeded in triplicate in 6-well plates (800 cells/well), allowed to adhere overnight, and incubated with media containing 10% FBS or human serum for 7 days. Cells were then fixed and stained with 0.05% methylene blue and colonies (>50 cells) were counted.

### 4.6. Immunoblotting

A549 cells were seeded in triplicate in 6-well plates (~100,000 cells/well), allowed to adhere overnight, and incubated with media containing either 10% FBS, pre-exercise, 5 min, 1 h, or 24 h post exercise serum for 30 min. Following the treatment, cell lysates were prepared, protein content was measured using the Biorad protein assay reagent, and samples (20 μg) were separated by sodium dodecyl sulfate-polyacrylamide gel electrophoresis (SDS-PAGE). Protein was then transferred to a polyvinylidene fluoride (PVDF) membrane for 1.5–3 h, blocked for 1 h with 5% skim milk, and incubated overnight with the indicated primary antibodies. The following day the membrane was incubated with horseradish peroxidase (HRP)-conjugated secondary antibody for 1 h. Protein was visualized using a LI-COR C-Digit blot scanner (LI-COR Biosciences) and corresponding software. Ponceau S (Biorad) was used to confirm the total protein content and transfer efficacy. Densitometric analysis was performed using ImageJ software.

### 4.7. Serum Insulin Measurements

Serum insulin was determined using a commercial quantitative sandwich enzyme immunoassay, with a detection range from 15.6 to 500 pmol/L and a sensitivity of 0.3 pmol/L. The intra assay coefficient of variation (CV) was 8.5%.

### 4.8. Statistical Analysis

The results are expressed as the mean ± SEM of the indicated number of independent experiments. Repeated measures analysis of variance (ANOVA) with Tukey post-hoc tests were performed and statistical significance was assumed at *p* < 0.05 using Graphpad Prism 5 software.

## 5. Conclusions

In summary, we were able to show that serum collected from young men after acute exercise resulted in significant inhibition of lung cancer cell survival and proliferation in vitro which was associated with significant inhibition of phosphorylation/activation of Akt, mTOR, p70 S6K, and Erk1/2 levels compared to cells treated with serum taken pre-exercise. Our data suggest that post-exercise serum has anti-cancer properties in lung cancer and deserves further systematic investigation in animal models of cancer and in humans.
